# Personality Traits Are Associated with Research Misbehavior in Dutch Scientists: A Cross-Sectional Study

**DOI:** 10.1371/journal.pone.0163251

**Published:** 2016-09-29

**Authors:** Joeri K. Tijdink, Lex M. Bouter, Coosje L. S. Veldkamp, Peter M. van de Ven, Jelte M. Wicherts, Yvo M. Smulders

**Affiliations:** 1 Department of Internal Medicine, VU University Medical Centre, Amsterdam, the Netherlands; 2 Department of Philosophy, Faculty of Humanities, VU University, Amsterdam, the Netherlands; 3 Department of Epidemiology & Biostatistics, VU University Medical Centre, Amsterdam, the Netherlands; 4 Tilburg School of Social and Behavioral Sciences, Tilburg University, Tilburg, the Netherlands; Universidad de las Palmas de Gran Canaria, SPAIN

## Abstract

**Background:**

Personality influences decision making and ethical considerations. Its influence on the occurrence of research misbehavior has never been studied. This study aims to determine the association between personality traits and self-reported questionable research practices and research misconduct. We hypothesized that narcissistic, Machiavellianistic and psychopathic traits as well as self-esteem are associated with research misbehavior.

**Methods:**

Included in this cross-sectional study design were 535 Dutch biomedical scientists (response rate 65%) from all hierarchical layers of 4 university medical centers in the Netherlands. We used validated personality questionnaires such as the Dark Triad (narcissism, psychopathy, and Machiavellianism), Rosenberg's Self-Esteem Scale, the Publication Pressure Questionnaire (PPQ), and also demographic and job-specific characteristics to investigate the association of personality traits with a composite research misbehavior severity score.

**Findings:**

Machiavellianism was positively associated (beta 1.28, CI 1.06–1.53) with self-reported research misbehavior, while narcissism, psychopathy and self-esteem were not. Exploratory analysis revealed that narcissism and research misconduct were more severe among persons in higher academic ranks (i.e., professors) (p<0.01 and p<0.001, respectively), and self-esteem scores and publication pressure were lower (p<0.001 and p<0.01, respectively) as compared to postgraduate PhD fellows.

**Conclusions:**

Machiavellianism may be a risk factor for research misbehaviour. Narcissism and research misbehaviour were more prevalent among biomedical scientists in higher academic positions. These results suggest that personality has an impact on research behavior and should be taken into account in fostering responsible conduct of research.

## Background

Little is known about the psychology and personality of biomedical scientists. We like to think that scientists are open, eager to collaborate, self-confident, curious and creative [1]. However, there’s anecdotal evidence that this is not universally so [2]. Success in science requires publishing in high Impact Factor journals and acquiring research grants, all in a hypercompetitive climate. This may tempt scientists to rush into print, cut corners, exaggerate findings and overstate the importance of their research [3]. In addition, the so-called Dark Triad of personality, referring to Machiavellianism, psychopathy, and narcissism has been found to predict behaviors like abusive supervision and employee theft [4]. Self-esteem appears to be negatively associated with cheating, at least among students [5,6].

The influence of personality traits on scientific practice is understudied. However, a negative relationship between narcissism and cynicism on the one hand, and aspects of ethical decision-making in future scientists on the other has been demonstrated consistently. [7] As these traits have also been found to predict cheating in scholastic,[8] financial,[9] and work[10] settings, it is conceivable that they are associated with an increased likelihood of engaging in research misbehavior (fraud and Questionable Research Practices or QRPs). Research misbehavior has received substantial attention in the last decade [11]. There is increasing evidence that research misbehaviors, specifically QRPs, are relatively common, seriously impact the scientific process, and compromises the validity of scientific results [12].

Preliminary data indeed suggest that specific personality characteristics are indeed associated with scientific misbehavior. Psychiatrist Kornfeld[13] distinguished different categories of fraudulent scientists, suggesting that certain personality profiles are more common among fraudsters, and found a relationship between publication pressure and research misbehavior. This qualitative evidence was supported by quantitative results [14].

In this study we aimed to provide more insight into the psychology of research misbehavior. We hypothesized that some scientists are more susceptible to committing research misbehavior than others. Specifically, we postulated that high self-esteem, Machiavellianism (a person’s tendency to be unemotional, detached from conventional morality and hence inclined to deceive and manipulate others, to focus on unmitigated achievement, and to give high priority to their own performance),[15] narcissistic traits (a person’s tendency to pursue gratification from vanity or egotistic admiration, and to obtain recognition of their own attributes),[16] and psychopathic traits (enduring antisocial behavior, diminished empathy and remorse, and disinhibited or bold behavior)[17], are associated with research misbehavior. The Dark Triad questionnaire with the 3 specific character traits (narcissism, Machiavellianism and psychopathy) was chosen to analyze the influence of these specific traits on research behavior. We hypothesized that narcissism, psychopathy and Machiavellianism could all play a role in an individual’s susceptibility to commit research misbehavior. If you are ‘bad’ in terms of dark personality traits, you are more likely to engage in bad behavior such as research misbehavior.

We hypothesized that publication pressure and academic position were mediating and possibly moderate/modify the effect of personality traits on research misbehavior; the higher the scores on the Dark Triad, the higher the scores on the research misbehavior sum score. More profound personality traits may, however, lead to more publication pressure, which in turn may result in more misbehavior (mediation). More profound personality traits may also make it more likely that someone occupies a higher academic rank, which may be related to more misbehavior (mediation).

Alternatively, more pronounced personality traits may be more strongly associated with research misbehavior in case of high academic position (moderation) due to researchers in top positions being more focused towards success in research. More pronounced personality traits may also be more strongly associated with research misbehavior in case of high publication pressure (moderation).

**Fig 1:**
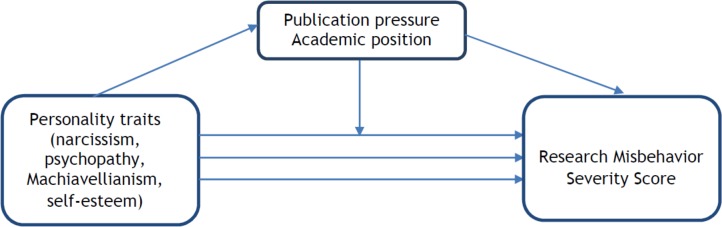
Predefined analysis model.

Conversely, we hypothesized that self-esteem could be beneficial for responsible conduct of research; if you feel confident in your job you have fewer reasons to misbehave. We therefore included self-esteem as a character trait in our predefined analysis model. (Fig 1)

In this study we furthermore wanted to determine whether publication pressure and academic position influence the relation between personality traits and research misbehavior.

## Methods

The medical Ethics Review Committee of VU University reviewed the study protocol and decided that the Medical Research Involving Human Subjects Act (WMO) does not apply to our study and that an official approval of our study by this committee is therefore not required.

### Participant selection and procedure

All 1833 biomedical scientists from 4 university medical centers in the Netherlands were invited by email to participate in the survey. Scientists were eligible if they were sufficiently proficient in English (as the questionnaires were in English), were scientifically active (studying research misbehaviors is relevant only for active scientists), and provided consent by following the link to the online questionnaire.

The research councils (the departments in charge of the policy of research-related matters within an institution) were essential in providing the e-mail addresses of potential participants in the 4 participating medical centers and inviting the participants. Two preclinical, three clinical (internal medicine, surgery, and psychiatry) and two supportive departments (i.e. methodology, statistics) were selected. To create heterogeneity among the participants we also included the most and least publishing departments per fte. The invitation e-mail (See Text A in S1 File) explained the objective of the study, using neutral terms such as achievement, motivation, personality and scientific success, and provided a link to an anonymous online questionnaire (Text B in S1 File) on a protected website. The e-mail also included the name and a link to the e-mail address of the lead investigator (JT) to opt out of participation.

Scientists who did not respond within 2 weeks were sent two reminders. After the second reminder we asked invited participants who still did not respond to fill out a 15 second ultra-brief questionnaire to determine their reason for declining participation.

### Psychometric information on questionnaires

The survey consisted of seven validated questionnaires (see Text B of S1 File) and a set of demographic questions. We used the Dark Triad [18] to measure Machiavellianism, narcissism and psychopathy. The Dark Triad consists of 27 items (9 items per subscale) on which participants indicate agreement on a five-point Likert scale, ranging from ‘strongly disagree’ to ‘strongly agree’. This scale is reliable (Cronbach’s α = 0.77–0.80). We used the Rosenberg Self-Esteem scale [19] to measure self-esteem. It consists of 10 items for which agreement is indicated on a four-point Likert scale, with answer options ranging from ‘strongly agree’ to ‘strongly disagree’. The Rosenberg Self-Esteem scale is also reliable (α = 0.77–0.88). To measure publication pressure we used the validated 14-item publication pressure questionnaire (PPQ) [20]. The biomedical scientists indicated to what extent they agree with each of the 14 statements, answer options ranging from ‘strongly disagree’ to ‘strongly agree’. Reliability of this scale is moderate (Lambda-2 reliability = 0.69).

For the primary outcome we constructed a research misbehavior (fraud and QRPs) severity questionnaire, which yielded a composite research misbehavior severity score. This questionnaire was based on questionnaires used by other research misbehavior investigators (see following cited references), with additional items gathered from different landmark publications on research misbehavior [12,14,21]. Based on these previous reports, we selected items deemed most suitable for biomedical scientists. The questionnaire consisted of 22 different types of research misbehaviors. Respondents were asked to report to what extent they had committed specified types of research misbehaviors the past three years. Answers were given on a 5-point scale (never, once, occasionally, frequently, often). To construct a Research Misbehavior Severity Score (RMSS), the scores of the items were dichotomously translated (behavior yes/no) and items were assigned different weights. The most severe type of misbehavior (based on the definition of fraud, i.e. fabrication, falsification and plagiarism) was 3 points. The other item scores were based on consensus in the research group and were assigned 1 (for moderate) and 2 (for severe) misbehavior. Positive answers (committing the behavior at least ‘once’ in the past 3 years) to the most severe misbehavior questions (items 1, 2, 8, 9, 12, 15 and 19) were assigned three points, positive answers of the severe research misbehavior questions were assigned two points (items 4, 7, 10, 14, 16, 18 and 20) and positive answers to the moderate research misbehavior questions were assigned one point (items 3, 5, 6, 11, 13, 17, 21 and 22). Scores were added up to calculate the composite research misbehavior severity score (RMSS) (maximum range: 0–43).

### Survey characteristics and outcomes

In Fig 1 we present the predefined analysis model. We postulated that 4 traits (narcissism, Machiavellianism, psychopathy, and self-esteem) are related to the outcome variable (RMSS). We hypothesized that this relation could be modified/moderated or, alternatively, mediated by publication pressure or academic position. To measure publication pressure we used the validated publication pressure questionnaire (PPQ) [20]. Academic position was operationalized by self-reported rank (postgraduate PhD-fellows, postdoctoral researchers, assistant professors, associate professors, and full professors).

Respondents provided demographic information on gender, age, academic position, type of biomedical specialty, number of years working as a scientist, percentage of time spent on research and (if known) Hirsch-index.

### Statistical analysis

Associations between personality traits and the outcome measure (RMSS) were first tested by means of linear regression analyses using a separate regression model for each personality trait. In order to satisfy the normality assumption for the residuals the outcome variable (RMSS) was transformed and log(1+RMSS) was used as the dependent variable. Personality traits were standardized by subtracting their sample mean and dividing by the standard deviation. The exponentiated regression coefficients refer to the relative increase of geometric means of RMSS+1 associated with an increase of 1 standard deviation on the personality trait scale. Effect modification/moderation was assessed by including the candidate modifier (publication pressure or academic position) in the regression model together with its interaction with the personality trait at issue. A variable was considered to be an effect modifier/moderator if it showed a significant interaction (p<0.05) with the personality trait. To assess whether publication pressure and academic position mediated the relationship between personality traits and scientific misbehavior we divided the total effect into a direct effect of the personality trait and an indirect effect via the candidate mediator. A variable was considered a mediator if both the total effect and the indirect effect were significant. In the mediation analyses for academic position we used a probit-link to relate academic position to the personality trait. To assess mediation we used a path model which regressed the mediator on the personality trait and then regressed the (transformed) RMSS score on the mediator. The coefficients of these two regression equations quantified the indirect effect from personality to RMSS score via the mediator. In addition, the path models directly regressed the RMSS score on the personality trait. The total effect was estimated in a model where RMSS score was regressed on the personality trait.

To assure validity of the analyses residuals from the models were checked for normality by means of visual inspection of histograms and QQ-plots.

In a secondary analysis, ANOVA tests were used to compare mean scores for personality traits, self-reported research misbehavior and publication pressure between respondents with different academic positions. In case of a significant overall difference, pairs of groups were compared in post hoc analyses using a Bonferroni correction.

To analyze the correlates of ethical behavior we performed a secondary post hoc analysis where we used logistic regression to test for associations of personality traits, PPQ and academic position with an RMSS of 0 (vs. RMSS > 0).

Regression analyses and ANOVA tests were performed in SPSS version 22. Analyses of mediating variables were performed in M-Plus version 7 using the product of coefficients methods.

### Survey statistics

In total, we used 1833 email addresses. Of these, 182 bounced because the email addresses no longer existed or were inactive. Of the remaining 1651, 1098 invitees opened the email. Among them, 715 started the survey (response rate 65%) and 537 completed the survey (completion rate 49%). We excluded 2 participants who declared they were not scientifically active. The demographic data of the complete responders are summarized in Table 1.

**Table 1:** Demographics.

		N = 535	%
Gender	Male	229	42.8%
	Female	306	57.2%
Age	<40	396	74%
	>40	139	26%
Academic Position	Postgraduate PhD-fellows student	303	56.6%
	Postdoc, Assistant or Associate professors	177	33.1%
	Full Professor	55	10.3%
Years working as a scientist	0–4	220	41.1%
	5–10	158	29.5%
	11–15	46	8.6%
	16–20	35	6.7%
	21–25	26	4.7%
	>25	49	9.2%

## Results

### Research Misbehavior Severity Score (RMSS)

The items of the research misbehavior questionnaire are tabulated in Table 2. The results of item 11 serve as an example of which items are included in the severity score: Almost 60% of the participants admitted having added authors who had made no significant contribution to the author list at least once. Fabrication and falsification were less common, although almost 5% admitted they had selectively deleted data to confirm a hypothesis. The median RMSS score in the sample was 3 (range 0–39).

**Table 2:** Items of the RMSS.

Research Misbehaviour item (N = 535)	0 times (%)	Once (%)	Several times (%)	Regularly (%)	Always (%)	Mean (SD)
1. Modified the results or conclusions of a study under pressure from an organization that (co-)funded the research?***	520 (97.2)	9 (1.7)	6 (1.1)	0	0	0.03 (0.32)
2. To confirm a hypothesis, selectively deleted or changing data after performing data analysis? ***	510 (95.3)	18 (3.4)	7 (1.3)	0	0	1.06 (0.29)
3. Deleted data before performing data analysis? *	473 (88.4)	24 (4.5)	32 (6.0)	4 (0.7)	2 (0.4)	1.20 (0.61)
4. Concealed results that contradicted previous research you published?**	510 (95.3)	20 (3.7)	5 (0.9)	0	0	1.06 (0.27)
5. Used phrases or ideas of others without their permission?*	466 (87.1)	38 (7.1)	27 (5.0)	4 (0.7)	0	1.19 (0.55)
6. Used phrases or ideas of others without citation? *	464 (86.7)	33 (6.2)	32 (6.0)	5 (0.9)	1 (0.2)	1.22 (0.61)
7. Turned a blind eye to colleagues’ use of flawed data or questionable interpretation of data?**	420 (78.5)	61 (11.4)	48 (9.0)	4 (0.7)	2 (0.4)	1.33 (0.70)
8. Fabricated data?***	533 (99.6)	1 (0.2)	0	0	1 (0.2)	1.01 (0.19)
9. Not published (part of) the results of a study?***	446 (83.4)	49 (9.2)	36 (6.7)	4 (0.7)	0	1.25 (0.61)
10. Deliberately not mentioned an organization that funded your research in the publication of your study?**	531 (99.3)	0	4 (0.7)	0	0	1.01 (0.17)
11. Added one or more authors to a report who did not qualify for authorship (honorary author)?*	213 (39.8)	130 (24.3)	150(28.0)	39 (7.3)	3 (0.6)	2.04 (1.01)
12. Selectively modified data after performing data analysis to confirm a hypothesis?***	514 (96.1)	16 (3.0)	5 (0.9)	0	0	1.05 (0.25)
13. Reported a downwardly rounded p value (e.g. reporting that a p value of .054 is less than .05)?*	524 (97.9)	7 (1.3)	3 (0.6)	1 (0.2)	0	1.03 (0.23)
14. Reported an unexpected finding as having been hypothesized from the start? **	429 (80.2)	63 (11.8)	39 (7.3)	4 (0.7)	0	1.29 (0.63)
15. Decided whether to exclude data after looking at the impact of doing so on the results?***	443 (82.8)	54 (10.1)	37 (6.9)	1 (0.2)	0	1.24 (0.58)
16. Decided to collect more data after seeing that the results were almost statistically significant?**	387 (72.3)	69 (12.9)	66 (12.3)	11 (2.1)	2 (0.4)	1.45 (0.82)
17. Omitted a contributor who deserved authorship from the author's list?*	521 (97.4)	7 (1.3)	6 (1.1)	1 (0.2)	0	1.04 (0.27)
18. Stopped collecting data earlier than planned because the result at hand already reached statistical significance without formal stopping rules?**	511 (95.5)	15 (2.8)	5 (0.9)	3 (0.6)	1 (0.2)	1.07 (0.38)
19. Deliberately failed to mention important aspects of the study in the paper?***	516 (96.4)	14 (2.6)	4 (0.7)	1 (0.2)	0	1.05 (0.27)
20. Not disclosed a relevant financial or intellectual conflict of interest?**	527 (98.5)	5 (0.9)	2 (0.4)	1(0.2)	0	1.02 (0.20)
21. Spread results over more papers than needed to publish more papers (‘salami slicing’)?*	440 (82.2)	53 (9.9)	29 (5.4)	13 (2.4)	0	1.28 (0.68)
22. Used confidential reviewer information for own research or publications?*	516 (96.4)	15 (2.8)	3 (0.6)	1 (0.2)	0	1.04 (0.26)

*Moderate misbehavior

** severe misbehavior

*** most severe misbehavior

### Machiavellianism predicts research misbehavior

Tables 3 and 4 present relations of personality traits with the research misbehavior severity score. Higher scores on Machiavellianism were significantly associated with higher research misbehavior severity scores. There was a trend (0.05≤p≤0.10) for narcissism and psychopathy having a similar association with research misbehavior. In multivariate analysis that included all three personality traits, only Machiavellianism was significantly associated with the RMSS (data not shown).

**Table 3:** Mediation analyses. Exponentiated regression coefficients, 95% confidence intervals and p-values for total, direct and indirect effects associated with 1 standard deviation increase in the personality trait.

		Mediation analysis			
		PPQ		Academic position	
	Total effect	Indirect	Direct	Indirect	Direct
Narcissism	1.08 (CI 1.00–1.16) p = 0.06	1.00 (CI 0.99–1.02) p = 0.76	1.07 (CI 1.00–1.16) p = 0.06	1.04 (CI 1.01–1.06) p = 0.005	1.04 (CI 0.96–1.12) p = 0.33
Psychopathy	1.08 (1.00, 1.16) p = 0.05 (ns)	1.00 (CI 0.98–1.01) p = 0.70	1.08 (CI 1.00–1.17) p = 0.04	1.01 (CI 0.99–1.03) p = 0.27	1.07 (CI 0.99–1.15) p = 0.08
Machiavellianism	1.12 (CI 1.04–1.21) p = 0.003	1.01 (CI 1.00–1.03) p = 0.07	1.11 (CI 1.03–1.20) p = 0.007	0.99 (CI 0.98–1.01) p = 0.90	1.12 (CI 1.04–1.21) p = 0.004
Self esteem	0.98 (CI 0.91–1.06) p = 0.60	1.01 (CI 0,99–1.02) p = 0.23	0.98 (CI 0.90–1.05) p = 0.46	0.96 (CI 0.94–0.99) p = 0.004	1.02 (CI 0.94–1.11) p = 0.63

**Table 4:** Academic position and personality traits. Table 4 shows the mean values of the Dark Triad: (Machiavellianism, narcissism, and psychopathy), self-esteem and PPQ sum score and comparison of means between groups using an ANOVA. Kruskal-Wallis test used to compare RMSS sum scores between groups.

		Postgraduate PhD-fellows (n = 303)	Postdoctorals, assistant & associate professors (n = 177)	Full professors (n = 55)	ANOVA
					p-value
Determinants					
Self-esteem	18.4 (CI 18.0–18.7)	18.8 (CI 18.4–19.3)	17.9 (CI 17.4–18.5)	16.9 (CI 16.1–17.8)	0.001
Narcissism	25.2 (CI 24.9–25.6)	24.7 ((CI 24.2–25.2)	25.4 (CI 25.1–26.3)	26.5 (CI 25.5–27.5)	0.002
Machiavellianism	25.0 (CI 24.6–25.3)	24.8 (CI 24.4–25.3)	25.4 (CI 24.8–26.0)	24.0 (CI 22.3–25.1)	0.09
Psychopathy	18.2 (CI 17.8–18.5)	18.0 (CI 17.6–18.5)	18.3 (CI 17.7–18.9)	18.7 (CI 17.7–19.8)	0.46
	Primary outcome measure				
RMSS		3.6 (CI 3.1–4.1); 2 (IQR: 1–5)	4.9 (CI 4.1–5.7); 4 (IQR: 1–7)	6.4 (CI 4.8–8.0); 5 (IQR: 2–9)	<0.001
	Candidate effect modifier/moderator				
PPQ sum score	42.2 (CI 42.0–43.1)	43.1 (CI 42.4–43.8)	42.2 (CI 41.3–43.3)	40.4 (CI 38.5–42.3)	0.017

Publication pressure as measured with the PPQ and academic position failed to significantly modify/moderate the relationship between the personality traits and research misbehavior severity: neither showed significant interaction with any of the personality traits (see S1 Table).

Variance in misbehavior explained by personality traits was only small, with 1.6% of variance explained by Machiavellianism (R^2^ = 0.016). Models for moderation/effect modification by academic position had R^2^ values ranging between 0.042 and 0.053. Models for moderation/effect modification by publication pressure had R^2^ values ranging between 0.031 and 0.038. Similar R^2^ values were found for models assessing mediation (see S3 Table).

### Role of publication pressure and academic position

We considered whether publication pressure or academic position mediated the relation between the Dark Triad and RMSS scores. The results of the mediation analyses are displayed in Table 3. In all mediation analyses PPQ and higher academic position were both found to be positively associated with RMSS scores (all p <0.001). R^2^ values for mediation models obtained from Mplus are included in S3 Table.

### Personality traits and demographic factors

Table 4 provides the means of the measured personality traits, PPQ and primary outcome (RMSS) stratified for academic position. Mean scores on narcissism and self-esteem were different between academic positions. Post-hoc tests revealed that postdocs, assistant and associate professors, and professors all scored higher on narcissism (Bonferroni corrected p < 0.05 and p <0.01 respectively) and lower on self-esteem than postgraduate PhD-fellows (Bonferroni corrected p < 0.05 and p < 0.01 respectively). No significant differences in personality trait scores were found between postdocs and professors. Furthermore, the RMSS scores differed between the academic position groups and post-hoc tests found RMSS scores to be higher for professors and postdocs as compared to postgraduate PhD-fellows (Bonferroni corrected p < 0.01 and p<0.001 respectively). No significant difference in RMSS was found between postdocs and professors. Finally, the publication pressure questionnaire score was found to differ between respondents with different academic positions. Post-hoc tests revealed a significant difference in PPQ scores between postgraduate PhD-fellows and full professors (Bonferroni corrected p < 0.05), with professors reporting lower publication pressure.

In the secondary analyses of the correlates of ethical behavior we looked at factors associated with an RMSS of 0 and we found that RMSS = 0 is more likely when PPQ is lower (OR 0.94 95% CI 0.91–0.97). There was a trend for academic position with higher academic position associated with RMSS > 0. (overall p = 0.053; postdoc compared with PhD-student OR 0.68 95% CI 0.43–1.09 and professor compared to PhD OR 0.39 95% CI 0.16–0.96). See S2 Table.

## Discussion

To our knowledge, this is the first study to investigate personality traits in relation to research misbehavior among biomedical scientists. Our results suggest that Machiavellianism is associated with self-reported research misbehavior. Secondary analyses reveal that narcissismand research misbehavior are positively associated with higher academic rank. Furthermore, unlike lower academic positions, higher academic positions are associated with both lower publication pressure and lower self-esteem.

Although evidence from earlier research is lacking, our results seem largely in agreement with the qualitative narrative analysis of Kornfeld [13], who gathered case histories of 146 individuals found guilty of research misbehavior and categorized them based on different psychological traits. According to Kornfeld, scientific fraud is the product of a combination of individual personality traits and an intense fear of failure, or the lure of academic and/or financial rewards [13]. Furthermore, most subjects indicated an intense pressure to publish as the main reason for their behavior, reasoning that publications boost their career potential and financial rewards. Our study is larger and non-selective and addresses a pre-defined hypothesis with quantitative data. In addition, Kornfeld’s study had no comparison group of non-fraudulent scientists. Also, personality traits in fraudsters who were caught may differ from those who are not (yet) caught.

In our analysis, Machiavellianism was associated with self-reported research misbehavior. Machiavellianism is best described as ‘a person’s tendency to be unemotional, detached from conventional morality and hence to deceive and manipulate others, to focus on unmitigated achievement and give high priority to own performances’[15]. This description intuitively explains that Machiavellianistic scientists more easily engage in research misbehavior. Moreover, the intellectual legacy of Niccolo Machiavelli confirms our findings as well. This is best illustrated by some of his quotes: ‘Whosoever desires constant success must change his conduct with the times’[22] and ‘One who deceives will always find those who allow themselves to be deceived’[23]. Comparison with existing literature on the general population revealed that the levels of the subscales of the Dark Triad (including Machiavellianism) in our study are comparable with the most recent literature[18], suggesting that Dark Triad traits are not stronger in biomedical scientists than in the general population.

Our secondary analyses suggest that narcissistic and psychopathic traits are more common in higher academic ranks and that scientists in higher academic ranks have less self-esteem. This could imply that the personality traits narcissism and psychopathy offer some kind of ‘survival benefit’ in academia. Whether these traits in higher academic ranks are desirable and ultimately will ultimately lead to the most ethical research behavior remain to be seen. Publication pressure was also lower in higher academic ranks, which is in line with earlier results[14].

To our knowledge, this is the first study to investigate personality traits and their relation with major and minor research misbehaviors among biomedical scientists. The response rate was high compared to other online surveys.[24] We chose anonymous questionnaires because of the potential relucatance of respondents to answer the questions on research misbehavior truthfully.[25,26]

The results of our study should, however, be interpreted cautiously. First, internet-based questionnaires can be influenced by response bias, e.g. by attracting participants who are not engaged in research misbehavior and reticence on the part of participants who have. To minimize this risk, we did not convey the purpose of this study in our invitational email and formulated the study goal in neutral terms (i.e. ‘We invite you to participate in this brief questionnaire that addresses personal characteristics of biomedical scientists in relation to science practice’, see Text A in S1 File). Furthermore, we asked participants who did not wish to participate to fill in an ultra-brief non-response survey. Of the 41 biomedical scientists who completed this survey, 56% cited lack of time, 22% cited that they were not involved in research and 10% cited the length of the questionnaire as the main reason for non-response. This together with the high response rate suggests that the findings may be generalizable to the total population of biomedical scientists in the Netherlands (and possibly the rest of the industrialized world).

The research misbehavior severity score should also be interpreted with caution. The 22 items are all self-reported, are prone to different interpretations and were measured at one time point only. The composite score should also be cautiously interpreted as we composed the score based on a self-designed (and thus arbitrary) one-dimensional score sheet and the input of earlier studies.

Taking these limitations into account, one should be hesitant in translating the results directly to the practice of, for example, hiring scientific personnel. However, they might have some implications for those who hire biomedical scientists in academia. One should be conscious about the possible influence of character traits on research behavior. Global assessment of the personality of applicants can certainly have some value in determining the right candidate for the job, but it should never play a central role in appointment procedures. It also can help increase awareness of certain personality traits in researchers and research groups. This awareness can help scientists gain more insight into and control over their behavior when they are in the middle of the scientific process. This insight might help individuals to become more aware of their misbehaviors and can ultimately reduce the risk and incidence of research misbehavior.

## Conclusion

Taken together the results suggest that, although narcissism and psychopathy may be associated with research misconduct at first sight Machiavellianism is the personality trait that is most strongly associated with research misbehavior. These results may inform those involved in the recruitment of scientific personnel, as well as people involved in scientific quality and integrity monitoring and those responsible for institutional research integrity policy and for responsible conduct of research training.

## Supporting Information

S1 Checklist (DOC)

S1 Dataset (SAV)

S1 File Invitational Email (Text A). Questionnaires included in the electronic survey (Text B).
(DOCX)

S1 Table Exponentiated regression coefficients Exp(beta) for linear regression of RMSS on personality traits and results of tests effect modification by PPQ and academic position (to clarify the beta scores: an increase of 1 standard deviation in Machiavellianism is associated with an increase of 12% in the geometric mean of RMSS+1). (DOCX)

S2 Table Table with subanalysis of RMSS = 0. (DOCX)

S3 Table Proportion of variance in outcome measure explained by the models. (DOCX)
