# Supplementary material for: Personality Traits Are Associated with Research Misbehavior in Dutch Scientists: A Cross-Sectional Study
Source: PLoS One. 2016 Sep 29;11(9):e0163251. doi: 10.1371/journal.pone.0163251 (PMC5042531; doi:10.1371/journal.pone.0163251)
Supplement: S1 Checklist — (DOC) [file pone.0163251.s001.doc]

STROBE Statement—checklist of items that should be included in reports of observational studies

|  | Item No. | Recommendation | | | | Page  No. |
| --- | --- | --- | --- | --- | --- | --- |
| **Title and abstract** | 1 | (*a*) The study’s design with a commonly used term is indicated in the abstract | | | | See abstract |
|  |  | (*b*) The abstract an informative and balanced summary of what was done and what was found is provided | | | | See abstract |
| Introduction | | |  | | | |
| Background/rationale | 2 | The scientific background and rationale for the investigation being reported has been explained | | | | Page 1 manuscript |
| Objectives | 3 | Specific objectives, including any prespecified hypotheses are stated | | | | Page 1 |
| Methods | | |  | | | |
| Study design | 4 | Key elements of study design early in the paper are presented | | | | Methods section, page 6,7 |
| Setting | 5 | The setting, locations, and relevant dates, including periods of recruitment, exposure, follow-up, and data collection are described | | | | Methods, Page 6,7 |
| Participants | 6 | We gave the eligibility criteria, and the sources and methods of selection of participants. | | | | Methods, page 6,7 |
| Variables | 7 | We have defined all outcomes, exposures, predictors, potential confounders, and effect modifiers. | | | | Methods, page 6,7 |
| Data sources/ measurement | 8* | For each variable of interest, we have given sources of data and details of methods of assessment (measurement). | | | | Methods, page 6,7 |
| Bias | 9 | We have described the efforts to address potential sources of bias | | | | Methods, page 6,7 |
| Study size | 10 | We have explained how the study size was arrived at | | | | Methods, page 6,7 |
| Quantitative variables | 11 | We have explained how quantitative variables were handled in the analyses. If applicable, describe which groupings were chosen and why | | | | Methods page 6,7 |
| Statistical methods | 12 | (*a*) We have described statistical methods, including those used to control for confounding | | | | Methods, page 7 |
|  |  | (*b*) We have described any methods used to examine subgroups and interactions | | | | Methods, page 7,8 |
|  |  | (*c*) We have explained how missing data were addressed | | | | Methods, page 7,8 |
| **Results** | | |  | | | |
| Participants | 13* | (a) We have reported the numbers of individuals at each stage of study—eg numbers potentially eligible, examined for eligibility, confirmed eligible, included in the study, completing follow-up, and analysed | | | | Results, page 10,11 |
| Descriptive data | 14* | We gave characteristics of study participants (eg demographic, clinical, social) and information on exposures and potential confounders | | | | Results, page 10 and table 1 |
| Outcome data | 15* | We have reported numbers of outcome events or summary measures over time | | | | Not applicable |
| Main results | 16 | 1. We have given unadjusted estimates and, if applicable, confounder-adjusted estimates and their precision (eg, 95% confidence interval). We made clear which confounders were adjusted for and why they were included 2. Report category boundaries when continuous variables were categorized N/A | | | | Results page 10,11 |
| Other analyses | 17 | We have reported other analyses done—eg analyses of subgroups and interactions | | | | Results page 11 |
|  | | |  |  | | |
| **Discussion** | | |  | | | |
| Key results | | | 18 | We have summarised key results with reference to study objectives | Discussion, page 12,13 | |
| Limitations | | | 19 | We have discussed limitations of the study, taking into account sources of potential bias or imprecision. Discuss both direction and magnitude of any potential bias | Discussion page 13,14 | |
| Interpretation | | | 20 | We gave a cautious overall interpretation of results considering objectives, limitations, multiplicity of analyses, results from similar studies, and other relevant evidence | Discussion page 13,14 | |
| Generalisability | | | 21 | We discussed the generalisability (external validity) of the study results | Discussion page 13,14 | |
| Other information | | |  | | | |
| Funding | | | 22 | We gave the source of funding and the role of the funders for the present study and, if applicable, for the original study on which the present article is based in the CoI statements | Additional information | |
