# Supplementary material for: Personality Traits Are Associated with Research Misbehavior in Dutch Scientists: A Cross-Sectional Study
Source: PLoS One. 2016 Sep 29;11(9):e0163251. doi: 10.1371/journal.pone.0163251 (PMC5042531; doi:10.1371/journal.pone.0163251)
Supplement: S1 File — Invitational Email (Text A). Questionnaires included in the electronic survey (Text B). (DOCX) [file pone.0163251.s003.docx]

**S1 File.** Invitational E-mail (Text A)

Subject: survey on personal characteristics of medical scientists

Dear scientist,

We are pleased to invite you to participate in this brief questionnaire that addresses personal characteristics of medical scientists in relation to science practice.

After agreement with the dean of your faculty, we received your name and e-mail address from the research council.

The survey is part of a larger research project involving publication culture and practice and this survey has been approved by both Ethics and other regulatory committees of the VUmc.

By clicking the link below, you can start of the survey, which is embedded in a fully protected website. The survey will take max.12 minutes of your precious time. Your responses will be completely anonymous.

https://onderzoekstool.nl/afname/start/?????

To encourage participation, 3 I-pads will be awarded to randomly selected participants. If you would like to participate in this raffle, please enter your e-mail address at the end of the survey.

Apologies for the unsolicited email and thanks in advance for your time. Your help would be extremely valuable.

On behalf of the research team,

Kind regards,

Joeri Tijdink, also on behalf of prof. dr. Y.M. Smulders

VU University Medical Center, Amsterdam

PS. If you decline participation, please let me know and I will delete your e-mail address from the mailing list. However, it would be great if you could briefly share your reason(s) for declining via e-mail in order to obtain more information regarding non-response: [j.tijdink@vumc.nl](mailto:j.tijdink@vumc.nl)

**S1 File.** Questionnaires included in the electronic survey (Text B).

Demographics

What is your gender?

- Male
- Female

What is your age?

- <25 years
- 25-35
- 36-45
- 46-55
- 56-65
- >65

What is your position?

- PhD-candidate
- Postdoctorate/MD-PhD
- Assistant professor
- Associate professor
- Professor (bijzonder hoogleraar)
- Full professor
- Medical doctor, not involved in research
- Other:

What is your specialty?

- (general) internal medicine
- surgery
- psychiatry
- microbiology
- public health
- pathology
- epidemiology
- methodology
- general practicioner
- clinical chemistry
- radiology
- dermatology
- other:

For how many years you are active in science?

- 0-4 years
- 5-10 years
- 11-15 years
- 16-20 years
- 21-25 years
- More than 25 years
- Other:

How much of your time you currently spend on research (in %)?

….%

What is your H-index?

- My H-index is:
- I don’t know
- I don’t know what a H-index is

***The Publication pressure questionnaire (PPQ)***

*This is a questionnaire analysing publication pressure. Please ﬁll in to what extend you agree on the next statements*

Likert Scale:

0 = totally disagree

1 = disagree

2 = no opinion

3 = agree

4 = totally agree

1. Without publication pressure, my scientific output would be of higher quality

2. My scientific publications contribute to better (future) medical care(R)

3. I experience my colleagues’ assessment of me on the basis of my publications as stressful

4. I experience the publication criteria formulated by my university for my appointment or re-appointment as professor as a stimulus(R)

5. Publication pressure puts pressure on relationships with fellow researchers

6. I suspect that publication pressure leads some colleagues (whether intentionally or not) to color data

7. The validity of medical world literature is increased by the publication pressure in scientific centers(R)

8. Publication pressure leads to serious worldwide doubts about the validity of research results

9. In my opinion, the pressure to publish scientific articles has become too high

10. The competitive scientific climate stimulates me to publish more(R)

11. My colleagues judge me mainly on the basis of my publications

12. Fellow scientists maintain their clinical and teaching skills well, despite publication pressure(R)

13. I cannot confide innovative research proposals to my colleagues

14. Publication pressure harms science

(R) reversed questions

**The Rosenberg Self Esteem Scale**

Below is a list of statements dealing with your general feelings about yourself. If you strongly agree, circle **SA**. If you agree with the statement, circle **A**. If you disagree, circle **D**. If you strongly disagree, circle **SD**.

SA= strongly agree, A= agree, D= disagree, SD= strongly disagree

1. In general I am satisfied with myself

2. Sometimes I think I am good for nothing (R)

3. I own several good qualities

4. I am capable of doing all sorts of things just as well as most other people

5. In my opinion I have not much to be proud of (R)

6. Sometimes I feel really useless (R)

7. I find myself just as much worth as others

8.I wish I had a little more selfrespect (R)

9. All things considerated I tend to call myself a loser (R)

10. I am fairly pleased with myself

(R) Reversed Items

**The Dark Triad**

The next questionnaire consists of twenty seven guidelines. Please rate your agreement or disagreement with each item using the following guidelines: If you strongly agree, circle **SA**. If you agree with the statement, circle **A**. If you neither agree nor disagree circle if you disagree with the statement, circle **D**. If you strongly disagree, circle **SD**.

1= strongly agree, 2= agree, 3=neutral 4= disagree, 5= strongly disagree

1. It's not wise to tell your secrets.
2. People see me as a natural leader.
3. I like to get revenge on authorities.
4. Generally speaking, people won’t work hard unless they have to
5. I hate being the center of attention. R
6. I avoid dangerous situations. R
7. Whatever it takes, you must get the important people on your side.
8. Many group activities tend to be dull without me.
9. Payback needs to be quick and nasty.
10. Avoid direct conflict with others because they may be useful in the future.
11. I know that I am special because everyone keeps telling me so.
12. People often say I'm out of control.
13. It's wise to keep track of information that you can use against people later.
14. I like to get acquainted with important people.
15. It's true that I can be mean to others.
16. You should wait for the right time to get back at people.
17. I feel embarrassed if someone compliments me. R
18. People who mess with me always regret it.
19. There are things you should hide from other people because they don't need to know.
20. I have been compared to famous people.
21. I have never gotten into trouble with the law. R
22. Make sure your plans benefit you, not others.
23. I am an average person. R
24. I like to pick on losers
25. Most people can be manipulated.
26. I insist on getting the respect I deserve.
27. I'll say anything to get what I want.

R = reversed item

**Research Misbehaviour Severity Score**

In your work as a scientist, have you shown, even if it has been only on a single occasion, any of the following behaviors in the last three years?

| 1. Modified the results or conclusions of a study under pressure from an organization that (co)funded the research? |
| --- |
| 1. To confirm a hypothesis, selectively deleted or changing data after performing data analysis? |
| 1. Deleted data before performing data analysis? |
| 1. Concealed results that contradicted previous research you published? |
| 1. Used phrases or ideas of others without their permission? |
| 1. Used phrases or ideas of others without citation? |
| 1. Turned a blind eye to colleagues’ use of flawed data or questionable interpretation of data? |
| 1. Fabricated data? |
| 1. Not published (important part of) the results of a study? |
| 1. Deliberately not mentioned an organization that funded your research in the publication of your study? |
| 1. Added one or more authors to a report who did not qualify for authorship (honorary author)? |
| 1. Selectively modified data after performing data analysis to confirm a hypothesis? |
| 1. Reported a downwardly rounded p value (e.g. reporting that a p value of .054 is less than .05)? |
| 1. Reported an unexpected finding as having been hypothesized from the start? |
| 1. Decided whether to exclude data after looking at the impact of doing so on the results? |
| 1. Decided to collect more data after seeing that the results were almost statistically significant? |
| 1. Omitted a contributor who deserved authorship from the author's list? |
| 1. Stopped collecting data earlier than planned because the result at hand already reached statistical significance without formal stopping rules? |
| 1. Deliberately failed to mention important aspects of the study in the paper? |
| 1. Not disclosed a relevant financial or intellectual conflict of interest? |
| 1. Spread results over more papers than needed to publish more papers (‘salami slicing’)? |
| 1. Used confidential reviewer information for own research or publications? |
