# Supplementary material for: Personality Traits Are Associated with Research Misbehavior in Dutch Scientists: A Cross-Sectional Study
Source: PLoS One. 2016 Sep 29;11(9):e0163251. doi: 10.1371/journal.pone.0163251 (PMC5042531; doi:10.1371/journal.pone.0163251)
Supplement: S1 Table — (DOCX) [file pone.0163251.s004.docx]

|  | | Effect modification | |
| --- | --- | --- | --- |
|  |  | PPQ | Academic position |
|  | Exp(beta)  95% CI  p-value | p-value  interaction | p-value  interaction |
| Narcissism | 1.08  (CI 1.00 - 1.16)  p = 0.06 | 0.70 | 0.07 |
| Psychopathy | 1.08  (CI 1.00 - 1.16)  p = 0.05 | 0.44 | 0.68 |
| Machiavellianism | 1.12  (CI 1.04 - 1.21)  p = 0.003 | 0.47 | 0.95 |
| Self esteem | 0.98  (CI 0.91 - 1.06)  p = 0.60 | 0.20 | 0.20 |

**S1 Table.** Exponentiated regression coefficients Exp(beta) for linear regression of RMSS on personality traits and results of tests effect modification by PPQ and academic position (to clarify the beta scores: an increase of 1 standard deviation in Machiavellianism is associated with an increase of 12% in the geometric mean of RMSS+1)
