# Supplementary material for: Personality Traits Are Associated with Research Misbehavior in Dutch Scientists: A Cross-Sectional Study
Source: PLoS One. 2016 Sep 29;11(9):e0163251. doi: 10.1371/journal.pone.0163251 (PMC5042531; doi:10.1371/journal.pone.0163251)
Supplement: S2 Table — (DOCX) [file pone.0163251.s005.docx]

| **Trait** | **OR (95% CI)** | **p-value** |
| --- | --- | --- |
| Self-esteem (per sd) | 1.09 (0.89, 1.35) | 0.410 |
| Narcism (per sd) | 0.86 (0.69, 1.06) | 0.145 |
| Macchiavellism (per sd) | 0.85 (0.69, 1.05) | 0.132 |
| Psychopathy (per sd) | 1.04 (0.84, 1.28) | 0.739 |
| Cynism (per sd) | 1.07 (0.86, 1.32) | 0.547 |
| PPQ (per 1 point increase) | 0.94 (0.91, 0.97) | <0.001 |
| Academic position |  | 0.053 (overall) |
| · Postdoc (compared to PhD) | 0.68 (0.43, 1.09) | 0.109 |
| ·  Professor (compared to PhD) | 0.39 (0.16, 0.96) | 0.039 |

**S2 Table.** Table with subanalysis of RMSS = 0.
