# Supplementary material for: Personality Traits Are Associated with Research Misbehavior in Dutch Scientists: A Cross-Sectional Study
Source: PLoS One. 2016 Sep 29;11(9):e0163251. doi: 10.1371/journal.pone.0163251 (PMC5042531; doi:10.1371/journal.pone.0163251)
Supplement: S3 Table — (DOCX) [file pone.0163251.s006.docx]

|  | **Model** | | | | |
| --- | --- | --- | --- | --- | --- |
| **Trait** | **Univariate linear regression** | **Moderation by Academic Position** | **Moderation by**  **Publication pressure** | **Mediation by Academic position** | **Mediation by Publication pressure** |
| Narcissism | 0.7 % | 4.8 % | 3.1 % | 3.4% | 3.0% |
| Psychopathy | 0.7 % | 4.3 % | 3.3 % | 5.3% | 3.2% |
| Machiavellianism | 1.6 % | 5.3 % | 3.8 % | 6.4% | 3.7% |
| Self-esteem | 0.1 % | 4.2 % | 3.2 % | 4.9% | 2.5% |

**S3 Table**. Proportion of variance in outcome measure explained by the models
